# Supplementary material for: Causal implicatures from correlational statements
Source: PLoS One. 2023 May 18;18(5):e0286067. doi: 10.1371/journal.pone.0286067 (PMC10194916; doi:10.1371/journal.pone.0286067)
Supplement: Attachment — Submitted filename: Causal implicatures response letter (1).pdf [file pone.0286067.s001.pdf]

Dear Dr. Goldwater,

Thank you for giving us the opportunity to revise our paper in response to these helpful comments from the reviewers. Below we respond to each comment. We would like to highlight two major changes:

- We report a new experiment which addresses some potential concerns about the first two experiments.
- We have expanded our discussion of the relevant psycholinguistic literature.

Best wishes,

Sam Gershman
Tomer Ullman

Reviewer 1

Overall, the paper is nicely and clearly written. The methods appear appropriately sound and the results are very clear with respect to the statistical hypotheses tested. I also commend the open materials and data though I confess I have not examined the data myself.

I have a few points to raise for revisions:

First, a question about one condition in Study 2 and its relation to Study 1. In Figure 1, the "association" condition in S2 appears to show chance responding. From the methods, I understand this condition was essentially the same as Study 1, which showed non-chance responding. Am I correct in interpreting this as a failure to replicate S1's results in S2? If so, I think it would be best for the authors to address this---S1 is preregistered and the effects observed are sizable enough I would expect them to replicate, so it seems a bit puzzling.

Thank you for raising this point, which we now address on p. 5:
"Although it is not entirely clear why this aspect of the data did not replicate Study 1, we suspect that it may be related to the context of more causally salient statements (risk increase/decrease, probability increase/decrease) which may have attuned participants to scrutinize association statements more carefully."

We also address this in the Discussion on p. 7:
"One potential concern about our findings is that the association condition produced different results in Studies 1 and 2, deviating from random in the former but not in the latter. We speculated that this might be due to some kind of context effect. Study 3 sheds some additional

light on this discrepancy, finding that most people endorse a non-causal interpretation of associative statements, although a large minority (47%) still favor a causal interpretation. Within that minority, we find again a preference for X → Y rather than Y → X. We tentatively propose that many or even most of the people endorsing Y → X in Study 2 were doing so as a signal of protest against being asked to assign a causal direction."

Next, I have two other concerns: one I feel is essential to any revision and another I think would help to improve the quality of the discussion.

First, I have a major concern about the correspondence between some of the claims made in the introduction and discussion and what the results actually seem to support. For instance, in the abstract, the authors write "We show that people do in fact infer causality from statements of association, under minimal conditions." But the conditions under which participants inferred causality were forced choice tasks requiring them to infer causality, asking only about the most likely direction. So it does not seem justified to claim that people "infer causality" on the basis of these findings. In the discussion, the authors write "... participants inferred a causal relation, such that the first variable causes the second." Again it does not seem justified to foreground the first part of that claim in this way. In both cases it would be better to hew closer to what was actually shown. Maybe something like "participants were biased to infer specific causal directionality," making clear that they did not spontaneously make causal inferences. I do recognize that the authors address this somewhat in the discussion, but the claims should be made consistent with the findings throughout.

Thanks for raising this point. We believe that the results of our new Study 3 (which includes a non-causal interpretation option) justify these strong claims: a majority of participants still endorse causal interpretations even when given the opportunity to endorse a non-causal interpretation (and thus are not required to report a particular causal direction).

Second, I think more could be done to clarify conceptually what is being claimed and to distinguish it from other (potentially deflationary?) explanations of the findings. The authors aim to test what they call their "causal implicature hypothesis." The implicature hypothesis is that "there are implicit causal expectations about linguistic structures that are activated by correlational statements, and these expectations can be made explicit when people are forced to choose between different causal interpretations".

As I understand it, the idea is that statistical correlation or "association" is a symmetric or non-directional relationship. So, if people's interpretations of these statements are really committed to this symmetry, then they should choose randomly in the forced choice causal-direction tasks ("If people truly are committed to non-causal interpretations of non-causal language, then participants should just choose arbitrarily between the different causal interpretations available to them.") Instead, the authors' studies have shown that, when pressed, people interpret such statements as implying a specific causal directionality. The logic of these studies is that observing symmetric

statements producing asymmetric interpretations gives evidence for the causal implicature hypothesis.

However, I see a few ways that the statements might not be seen as truly symmetric, especially in Study 2. Consider "increases risk of" statements: On the one hand it's true that if $P(A|B) > P(A)$, then $P(B|A) > P(B)$, but on the other hand the degree of the inequality might not be at all symmetric. For instance, some rare event A might greatly increase risk of B, yet A could still remain fairly unlikely given B. In such a case it would be literally true to say both that "A increases risk of B" and "B increases risk of A", but it might be pragmatically more informative to say "A increases risk of B". (I suppose whether this is so would depend on how people think about quantifying risk differences. In terms of mutual information things are symmetric, but in terms of differences like $P(A|B) - P(A)$ they need not be.) So statements of association can sometimes be asymmetric without any causal content. Participants might then be aligning their causal directionality with the "directionality" of the original statements.

Second, the effects might not be due to implicatures but rather something like vagueness. One possibility is people might more directly interpret the claims about association as being based on experimental evidence. They might interpret the statement "X is associated with increased risk of Y" as "_doing_ X is associated with an increased risk of Y". To be sure, pragmatically it is odd for a speaker to say "associated with an increased risk of" rather than "increases risk" or "causes" in such a case. But, participants are ultimately given a forced choice between two causal interpretations. When forced to pick a causal interpretation, it seems like the statements in Study 2 at least leave open the "doing" reading, and hence would support the causal interpretations participants made. (note also that these "doing" interpretations are asymmetric.)

To address this concern I'd like to see more careful discussion of how the authors think participants actually interpret the statements in their studies and how they might interpret such statements outside the lab. I could be convinced that the objections I've raised are not major problems for what I take to be the spirit of the authors' conclusions: e.g. as I said I think you could construe part of my complaint as a question about whether their results are due to pragmatic implicatures or simply vagueness. In practice, we might expect similar consequences and thus it seems reasonable to care about both: Presented with careful statements of association by scientists, lay people will (at least sometimes) interpret them as loose statements about causal relationships. And on the other front, if statements of association have inherent (pragmatic) asymmetry, which lead to causal implications, that seems worth knowing too. Still I'd encourage the authors to clarify their claims and contrast their causal implicature hypothesis with alternate accounts. I'd also encourage them to consider how different accounts of their findings then relate to real-world consequences (e.g. especially in relation to their forced-choice task---without these pressures misinterpretations might be less likely).

Thanks for raising these concerns, which helped us refine how we discuss our results. We have added several paragraphs to the Discussion addressing them:

“Our results cannot unambiguously rule out several alternative hypotheses that hinge on different interpretations of the information we provided to participants. First, it is possible that participants did not distinguish between probabilistic and causal interpretations of the statements. For example, the statement that X increases the risk of Y might also imply that Y increases the risk of X, but the increase for Y given X might still be larger than the increase for X given Y. In this case, there is an asymmetry in the risk pattern, even if there is no causal relationship between X and Y. If participants align their causal preferences with the directional asymmetry, then they might show preferences deviating from random, which we have treated as a signature of causal implicature. In essence, this alternative hypothesis posits either that participants do not fully understand what causality is, or that risk directionality is used as a heuristic for causal implicature. We think it is somewhat unlikely that participants simply do not understand what we are asking when we elicit causal interpretations, given the sophisticated causal reasoning abilities demonstrated in many other studies.

A second alternative hypothesis is that participants interpreted statements of the form ``X is associated with increased risk of Y'' as ``Doing X is associated with increased risk of Y'' which would seem to imply a kind of causal intervention. Thus, on this hypothesis apparent causal preferences arise from vagueness in the statements. While we cannot rule out this possibility, we would argue that the vagueness hypothesis is really another version of causal implicature, where participants make a pragmatic interpretation that the speaker intends to communicate a causal relationship.

We have argued that the alignment and vagueness hypotheses may or may not be consistent with a causal implicature hypothesis. More work is required to answer this question.”

Reviewer #2

Correlation does not imply causation and people are often warned of this fact. This suggests people may tend to ascribe causes even when purely correlational statements are made. This is the premise of the paper.

The paper is very brief and I'll keep my comments on it brief as well. Although the studies seem like reasonable first passes at studying the question, there is next to no discussion of the rich literature that exists and how different syntactic structures may prompt people to think an antecedent caused a consequent. Some of this work is cited. None of the details of these results are discussed to contextualize the studies that have been run.

In my view, this paper reads like a blog post---and that has some virtues--- but doesn't give enough to the reader enough to understand their findings, nor really engages with

the fact that people have thought about this and related questions before. For the work to be publishable, the authors will need to write a paper instead of a blog post.

Thanks for this suggestion. We agree that the coverage of the literature was too limited. We have expanded the Introduction to provide more context and to explain how our work is related to the literature on implicit causality.
