# Supplementary material for: Causal implicatures from correlational statements
Source: PLoS One. 2023 May 18;18(5):e0286067. doi: 10.1371/journal.pone.0286067 (PMC10194916; doi:10.1371/journal.pone.0286067)
Supplement: Attachment — Submitted filename: GershmanUllman Plos One response2.pdf [file pone.0286067.s002.pdf]

Dear Dr. Goldwater,

Thank you for giving us the opportunity to submit another revision of our paper.

There is just a single minor revision needed before the paper can be accepted. There is a journal policy (that I was recently reminded of) that there needs to be safeguards of data quality for online data sources such as from Prolific, which you used here. Given the rise of bots and AI tools to answer surveys, this has become even more critical in just the past six months. I see that "participants were restricted to those located in the USA, and having completed at least 100 prior studies on Prolific, with an acceptance rate of at least 90%." And further, they were asked "Please describe, in a few words, what you were asked to do in this experiment?”" at the end of the study. If there is further evidence to provide that when people automate responses, they are likely to be rejected (and so would not reach a 90% acceptance rate), and whether this last sentence would be difficult to automatically generate a response that actually reflected the content of the study, that would help increase the confidence in the data quality. I assume providing this further evidence will not be much of a burden.

We have now added a subsection to the Methods titled “Data quality assurance”:

We appreciate the growing concern about using AI tools to answer online surveys, and we do not think there is an agreed-on safeguard yet against the use of latest tools like ChatGPT-4 to answer online catch questions. We would note that the first two studies were run in early 2022, when the use of large language models was not widespread, and their performance when used was rather limited. Our catch question was meant to weed out low-effort, automatic responses such as `have a good day or `relationships'. To the degree that we did not weed out all auto-replies, we believe that they only introduced a bit of noise, and given that our studies report differential effects this extra noise on top of the main effect is not a primary concern. The third study was run in late 2022, around the advent of the (now legacy) version of ChatGPT, and before adjusting for how well (or not) such tools can pass various tasks. We cannot guarantee our last question weeds out malicious users of the latest automatic tools, and moving forward we plan to adjust our data validation. However, we believe that the timing of the study was such that it is unlikely a large amount of malicious users on Prolific (to the degree such a group existed) had time to mass adopt ChatGPT into their workflow and rack up hundreds of completed and approved studies in time for our study. We also note that the marginal financial gain of using these tools is likely outweighed by the cost of per-token expenses.
